# EXPLORING THE NEEDS OF LGBTQIA+ CAREGIVERS OF PEOPLE LIVING WITH NEURODEGENERATIVE DISEASES

**DOI:** 10.1093/geroni/igae098.0798

**Published:** 2024-12-31

**Authors:** Jason Flatt, Cody Yamada, Brittany Klenczar, Krystal Kittle, Whitney Wharton, Joel Anderson

**Affiliations:** University of Nevada Las Vegas, Las Vegas, Nevada, United States; University of Nevada Las Vegas, Las Vegas, Nevada, United States; University of Nevada Las Vegas, Las Vegas, Nevada, United States; University of Massachusetts Amherst, Springfield, Massachusetts, United States; Emory University, Atlanta, Georgia, United States; University of Tennessee-Knoxville, Knoxville, Tennessee, United States

## Abstract

The Administration on Aging estimates 4 million adults aged 60+ in the U.S. identify as lesbian, gay, bisexual, transgender, queer, intersex and/or another identity (LGBTQIA+). Experiences and health-related concerns of LGBTQIA+ caregivers of people living with a neurodegenerative disease, such as Alzheimer’s disease and/or Parkinson’s disease (AD/PD), have been understudied. We synthesize data from two qualitative studies with LGBTQIA+ AD/PD caregivers, including Parkinson’s Research with Inclusion, Diversity, and Equity (PRIDE) and the Research Inclusion Supports Equity (RISE) Registry. Interviews were conducted with LGBTQIA+ caregivers of people living with AD (n=25) and PD (n=8) to explore their needs, experiences, and concerns with using aging and long-term care services and personal health and social needs. Thematic analysis was used to identify key themes. The first theme involved LGBTQIA+ AD/PD caregivers experiencing several financial, legal, and social challenges (e.g., reluctance to access long-term care services) when caring for someone living with AD/PD. Another theme involved LGBTQIA+ caregivers describing that they often supported their care recipients with medical-/nursing-related tasks, stress from caregiving demands, and experiencing financial and/or eligibility challenges when accessing respite care. The final theme focused on caregivers’ health and access of services, with LGBTQIA+ AD/PD caregivers describing difficulties managing their own health, feelings of depression and loneliness, and perceived discrimination when accessing long-term care and aging-related services. We highlight the need for additional research and creation of innovative long-term care services and caregiver support programs to better meet the needs of LGBTQIA+ caregivers of people living with AD/PD.

